# Regulatory mechanisms of group distributions in a gregarious arthropod

**DOI:** 10.1098/rsos.150428

**Published:** 2015-11-25

**Authors:** Pierre Broly, Romain Mullier, Cédric Devigne, Jean-Louis Deneubourg

**Affiliations:** 1 Unité d’Ecologie Sociale, Université Libre de Bruxelles, Campus de la Plaine, Bruxelles, Belgium; 2 Laboratoire Ecologie et Biodiversité, Faculté de Gestion, Economie et Sciences, UCLILLE, Lille, France; 3 Forensic Taphonomy Unit, University Lille Nord de France—UDSL, 59000 Lille, France

**Keywords:** aggregation, patchy environment, scale-dependent feedbacks, local activation/long-range inhibition mechanism, group size

## Abstract

In a patchy environment, how social animals manage conspecific and environmental cues in their choice of habitat is a leading issue for understanding their spatial distribution and their exploitation of resources. Here, we experimentally tested the effects of environmental heterogeneities (artificial shelters) and some of their characteristics (size and fragmentation) on the aggregation process of a common species of terrestrial isopod (Crustacea). One hundred individuals were introduced into three different heterogeneous set-ups and in a homogeneous set-up. In the four set-ups, the populations split into two aggregates: one large (approx. 70 individuals) and one smaller (approx. 20 individuals). These aggregates were not randomly distributed in the arena but were formed diametrically opposite from one another. The similarity of the results among the four set-ups shows that under experimental conditions, the environmental heterogeneities have a low impact on the aggregation dynamics and spatial patterns of the isopod, merely serving to increase the probability of nucleation of the larger aggregation at these points. By contrast, the regulation of aggregate sizes and the regular distribution of groups are signatures of local amplification processes, in agreement with the short-range activator and long-range inhibitor model (scale-dependent feedbacks). In other words, we show how small-scale interactions may govern large-scale spatial patterns. This experimental illustration of spatial self-organization is an important step towards comprehension of the complex game of competition among groups in social species.

## 1. Introduction

An area can be fragmented at various scales, resulting in several subunits (i.e. heterogeneous patches) according to many biotic and/or abiotic factors [1,2]. Resource density and its availability for foraging, breeding or sheltering, according to the physical constraints of the environment (e.g. temperature and moisture content), are major sources of heterogeneity. Therefore, according to its intrinsic quality, each patch can offer various gains for individuals [3] and obviously leads to a non-random selection of habitats at the group or population level and to a non-homogeneous distribution of the individuals due to their similar response to these spatial characteristics. In addition, the individuals may choose between environmental heterogeneities according to social information [4–6], and these choices may be reinforced by an inter-attraction between individuals in social species, such that the more individuals there are, the more the patch is attractive [7–9]. Two extreme cases are described in the literature: cases where the inter-attraction cannot emerge without heterogeneity, such as trail recruitment in ant foraging [10] and when the inter-attraction is permanent and independent of the heterogeneities, such as aggregations of woodlice or cockroaches [11,12]. Such inter-attractions, at the basis of positive feedback loops, lead to clustering in a homogeneous or symmetrical world and to symmetry that breaks down when individuals are confronted with choices involving identical heterogeneities [7–9]. Furthermore, different effects, such as crowding, limitation of the resources or agonistic behaviours at high density, may act as negative feedback loops and limit the size of the clusters [13].

In this context, how gregarious species assess a patchy environment and respond collectively to its partitioned resources is a central question in ecology, especially because group size and group organization may lead to high variations in the *per capita* fitness of members [14–16]. The collective assessment of a patchy environment and exploitation of partitioned resources by gregarious species may emerge in various spatial patterns, including overcrowding effects and ideal free distribution [7,13,17–20]. In self-organized systems, theoretical models predict that the amplification of local cues may lead to regulatory mechanisms governing the spatio-temporal distribution of groups in the environment. However, few experimental demonstrations of such scenarios exist in social systems [15,18,21]. Yet, such a question is biologically relevant because pattern formation, especially regular patterns, may have important effects on ecosystem functioning [15,21,22].

Shelters are an important resource for many species that are unable to build or dig a refuge. These shelters, which are microhabitats, reduce environmental stresses such as predation or desiccation. In woodlice (Crustacea, Isopoda and Oniscidea), shelters are very important, especially for body water conservation. Woodlice are terrestrial crustaceans that are extremely sensitive to water loss [23,24] and must find moist shelters to survive between two nocturnal phases of foraging [25]. As a consequence, resting in shelters is the most observed daylight behaviour in woodlice [25,26]. The presence of conspecifics may be an important cue in the decision-making process when choosing between the multitudes of available resources, as proved by the inter-attraction between isopods confronted with the choice of two identical shelters [12,27].

In this study, we explore how the interplay between the environmental heterogeneities and the social mechanisms at work in group cohesion may govern the size and the spatio-temporal distribution of groups. For this, we performed aggregation experiments with a social species, a common species of terrestrial isopod, in a homogeneous set-up and in three heterogeneous set-ups, varying the number and the size of heterogeneities (shelter). Our hypothesis was that the environmental heterogeneities are of a lesser importance for the spatio-temporal distribution of individuals than the social interactions. Our results show that the individuals respond positively to environmental heterogeneities. However, the social mechanisms underlying the aggregation behaviour in this species predominantly lead to the regular distribution of aggregates and a regulation of their size. In this respect, we show how small-scale interactions may govern the self-organization of large-scale spatial patterns.

## 2. Material and methods

### 2.1 Biological material

Several hundred specimens of the isopod species *Porcellio scaber* (Latreille, 1804) were captured in the gardens of the Catholic University of Lille (Northern France) and in a deciduous forest of the Belgian Ardennes. Individuals from the two localities were placed together and maintained in captivity in closed terrariums (410×240×225 mm). The layer of moistened plaster lining the back of the terrariums maintained a high humidity (more than 80%). Temperature varied from 21.5°C±2°C and the natural photoperiod in the region was respected. Woodlice were fed with leaf litter (primarily maple leaves).

### 2.2 Set-up diversity

**Figure 1.**
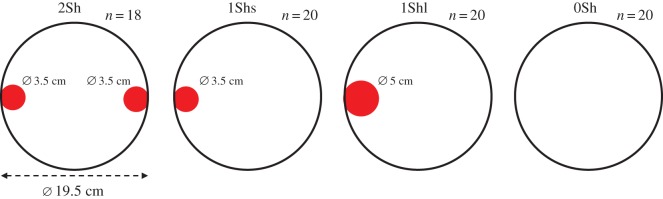
Schematic of the four experimental set-ups.

Four set-ups were designed (figure 1). All have a basic model: a uniform and circular PVC arena (19.5 cm diameter) lighted with a brightness of 166 lux. The bottom of the set-up consists of a sheet of white paper that is replaced between each experiment.

The first set-up (2Sh—two small shelters) was made with the basic arena described above with the addition of two shelters. These shelters consisted of round pieces of glass (3.5 cm diameter), stuck to the wall of the arena (0.5 cm above the ground) and diametrically opposite one another in the set-up. These shelters were covered with two layers of red filter (ROSCO® ref. Roscolux Fire # 19); thus, the brightness of 166 lux in the arena declines to 41 lux under the shelter. For this condition, *n*=18 trials were done.

The second set-up (1Shs—one small shelter) was built on the same model as the 2Sh device but contained only one shelter of 3.5 cm diameter. For this condition, *n*=20 trials were done.

The third set-up (1Shl—one large shelter) was identical to set-up 1Shs but the unique shelter of 3.5 cm diameter was replaced by a larger shelter (Ø 5 cm). The surface of this large shelter (20 cm^2^) was double the size of the surface of a small shelter of 3.5 cm diameter (10 cm^2^). The contact surface favourable to thigmotactism is also doubled (see the electronic supplementary material, figure S1; 1 cm versus 2 cm) so that the large shelter of 5 cm diameter is, in all points, a double shelter of 3.5 cm diameter. In other words, this shelter offers the same hiding capacity of the 2Sh device, but the heterogeneity is not fragmented. For this condition, *n*=20 trials were done.

The fourth and last set-up (0Sh—no shelter) consisted only of an arena without the shelter described previously (i.e. basic model). This set-up constituted a homogeneous set-up. For this condition, *n*=20 trials were done.

In each set-up, 100 woodlice, previously held undisturbed in a central removable arena (65 mm diameter; see methodology in [28]), were released into the arena, and the aggregation process was followed for 45 min by video recording (Sony camera CCD firewire—DMK 31BF03).

Experiments of each condition were carried out in daytime from January to May in an inter-condition mixed order throughout the experimental period.

### 2.3 Measures and statistical analysis

An aggregate was considered to be a gathering of two or more woodlice in contact for at least 2 min. Each aggregate was followed from its emergence to its dispersion, during which the number of woodlice inside were counted each minute.

Several aggregations may form in the set-ups during the experiments. To distinguish them, in each experiment, they were ranked according to their mean size (mean number of individuals inside during their lifetime) as follows: first aggregate (the biggest aggregate), second aggregate (smaller than the first aggregate and larger than the third), third aggregate…*n*^*th*^ aggregate (the smallest). The aggregates from the second place and following were pooled under the term ‘secondary aggregates’ in contrast to the principal aggregate (first).

To measure the surface area of the aggregates under shelter, a picture was taken at the end of each experiment, and the number of pixels occupied by the aggregate was counted with Photoshop 7.0.1 (Adobe Systems Software). The area per individual represents the total surface of the aggregate divided by the number of individuals included in the aggregate.

The measure of the angular position of the aggregates was taken from the centre of the aggregate mass. The angular distribution of the secondary aggregates was measured from the normalized position of the first aggregate (0°).

The statistical tests, figures and regression analyses were performed using GraphPad Prism v. 5.01 (GraphPad Software Inc.) and R v. 3.1.2. Kernel density representations were obtained with PAST v. 2.17 using Cartesian coordinates of individuals recorded with the software Regressi and its plug-in Regavi (Micrelec, France).

## 3. Results

### 3.1 General patterns and aggregation dynamics

**Figure 2.**
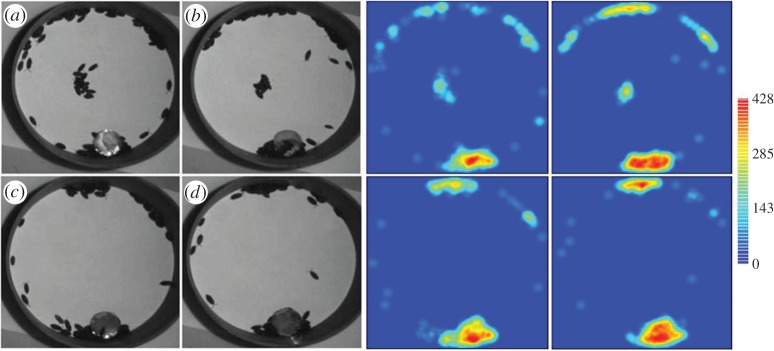
An example of aggregation dynamics in the arena with one shelter of ø 3.5 cm and 100 woodlice at *t*=5 min (*a*), *t*=10 min (*b*), *t*=30 min (*c*) and *t*=45 min (*d*), and its representation using Kernel density on the right side (paraboloid function). See other examples in the electronic supplementary material, figure S2.

**Figure 3.**
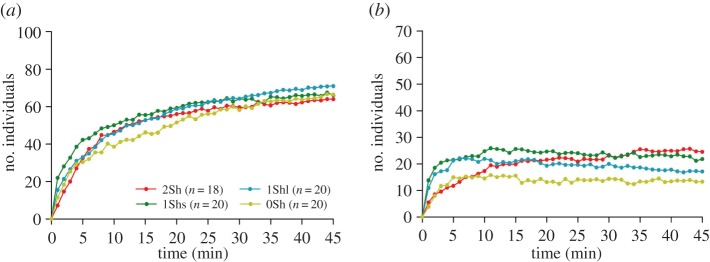
Mean dynamic of aggregation (number of woodlice as a function of time) in the first (*a*) and second (*b*) aggregate. Colours indicate the experimental condition.

As a common trend, the introduced populations quickly split up into several aggregates at the periphery of the arena during the first few minutes of the experiment (e.g. figure 2; electronic supplementary material, figure S2). All experiments presented at least one aggregate during the 45 min. A complete analysis of the dynamics of the aggregation process in the different aggregates is given in the electronic supplementary material, figure S3. Briefly, the global dynamic of aggregation (cumulative aggregates) was similar among the four set-ups and reached a plateau at approximately 80–90 aggregated individuals at the end of the experiments (table 1; electronic supplementary material, figure S3*a* and S3*b*). There is an inter-set-up difference in the total number of aggregates formed during the experiment (more aggregates are formed in the set-up without shelter than those including two shelters; Kruskal–Wallis test and Dunn post-test; KW=15.875, d.f.=3, *p*=0.0012; electronic supplementary material, figure S3*c*), but there is no inter-set-up difference at the end of the experiments (Kruskal–Wallis test, KW=5.947, d.f.=3, *p*=0.1142). In more than 75% of the experiments, only two aggregates remained at the end of the experiment (table 1; electronic supplementary material, figure S3*c*), and these aggregates differed significantly in size in more than 75% of the cases (table 1; binomial test, *p*<0.001). The dynamics of aggregation were fast in the first and second aggregate and stabilized, at approximately 70 individuals (figure 3*a*) and 20 individuals (figure 3*b*), respectively. At the end of experiments, there was no inter-set-up difference in the number of individuals included in the first aggregate (Kruskal–Wallis test, KW=3.176, d.f.=3, *p*=0.3635; electronic supplementary material, figure S3*d*) and in the second aggregate (Kruskal–Wallis test, KW=7.642, d.f.=3, *p*=0.0654; electronic supplementary material, figure S3*e*). The other secondary aggregates (third or more aggregate) presented a particularly weak population size, stability and lifetime (electronic supplementary material, figures S3 and S4).

**Table 1.** Summary of some components of the aggregation in the four set-ups at the end of experiments (except for the mean number of individuals in the first aggregate, the data are calculated for the entire lifetime of the aggregate). The significant difference between the size of the first and second aggregate is obtained through a binomial test.

	total number of woodlice aggregated (s.d.)	average number of aggregates (s.d.)	percentage of first aggregate under a shelter	mean number of individuals in the first aggregate (s.d.)	mean number of individuals in the second aggregate (s.d.)	experiments with a difference in size between the first and second aggregate (%)
2Sh (*n*=18)	88.7±6.76	1.89 (0.32)	100	64 (13.4)	21.89 (11.7)	78
1Shs (*n*=20)	89.4±5.58	2.05 (0.68)	90	66.1 (18.2)	22.78 (14.34)	80
1Shl (*n*=20)	93.05±6.66	2.4 (0.82)	95	71.9 (15.9)	19.4 (13.93)	100
0Sh (*n*=20)	82.4±10.29	2.2 (0.95)	—	66.4 (14.5)	13.09 (8.79)	80

**Figure 4.**
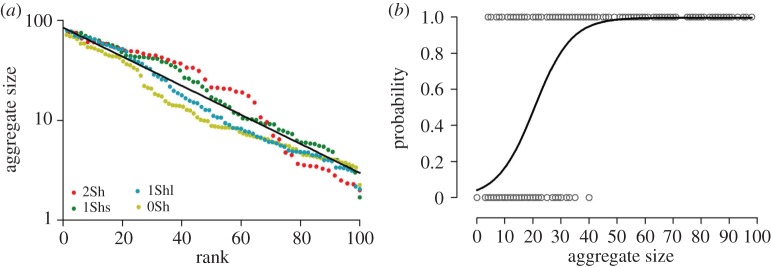
(*a*) Distribution of aggregate size (mean number of individuals included in the aggregate during its lifetime) during the experiments according to the set-up. Logarithmic scale for the *y*-axis. The solid line represents the mean exponential fitting of the pooled data *Y* =*a*.*e*^−*bX*^ with *a*=85.1 (±0.565) and *b*=−0.033 (±0.000319) (d.f.=270; *R*^2^=0.9876). (*b*) Probability that an aggregate will be present (=1) or dislocated (=0) at the 45th minute according to the maximum size (maximum number of aggregated individuals inside) reached during the experiment. The data are fitted by a logistic function *y*=*a*/(1+*e*^−*c*(*x*−*T*)^) (*R*^2^=0.4616).

The distribution of the mean aggregate size decreases similarly according to the set-up with an exponential function (figure 4*a*), also some quantitative differences may be found between the set-ups (*F* test, *F*=92.4, d.f.=2, *p*<0.0001). In addition, the probability that an aggregate will survive (i.e. the probability that it will not dislocate during the experiment) increases with aggregate size according to a logistic function:=*a*/(1+*e*^−*c*(*x*−*T*^)), with *a*=0.997, *c*=0.152 and a threshold value *T*=20.623 (d.f.=270; *R*^2^=0.4616; figure 4*b* and see also the electronic supplementary material, figure S4). Therefore, the probability that an aggregate will survive is greater than 0.5 when the aggregate size is greater than 20 individuals (figure 4*b*).

### 3.2 Spatial conformation of aggregates

A complete analysis of the spatial conformation of aggregated individuals is given in the electronic supplementary material, figure S5. Briefly, the aggregates, either contained in a set-up with shelter or not, present the same spatial spreading and the same area per individual for the first and the second aggregates (electronic supplementary material, figure S5*a* and S5*b*). Only the number of individuals included in the aggregate depressed the surface area per individual (electronic supplementary material, figure S5*c*). In addition, the shelters of 3.5 cm diameter were full at the end of experiments (more than 90% of their total carrying capacity is occupied by the aggregates; electronic supplementary material, figure S5*d*), which was not the case for the large shelter of 5 cm diameter (only 60% full; electronic supplementary material, figure S5*d*). However, the aggregates may overflow the shelter limits (up to 40% of the total surface area of the aggregates; electronic supplementary material, figure S5*e*).

### 3.3 Localization of the aggregates

In the set-ups that included one or two shelters, the first aggregate was always formed under a shelter (table 1), except in only three experiments of 58 where the first aggregate was outside the shelter, whereas the shelter gathered a smaller aggregate (two in 1Shs and one in 1Shl). In the set-up without a shelter, the first aggregate was always formed on the edge of the arena and randomly distributed in the arena (i.e. no favoured angular distribution was apparent; Rayleigh test, *R*=0.3055, *p*=0.1553).

**Figure 5.**
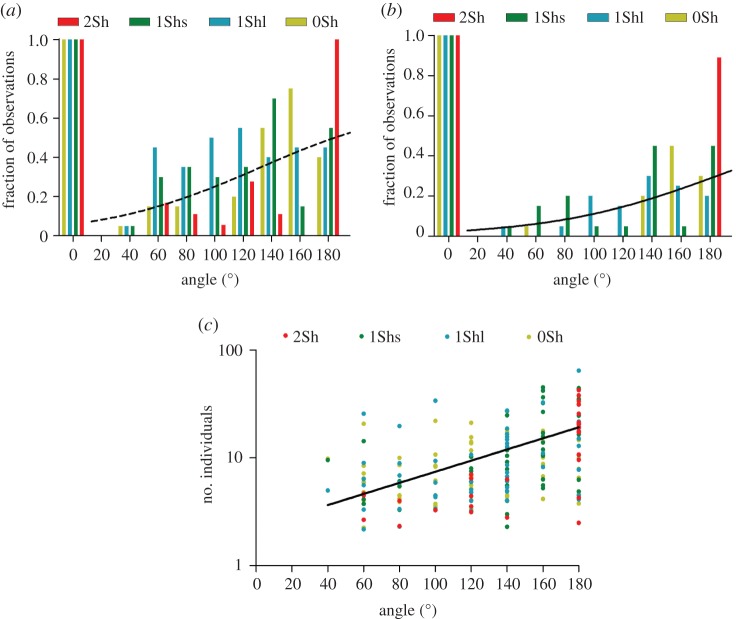
(*a*) Angular distribution of the aggregates during the experiments. The value 0° is the normalized position of the first aggregate. The dotted line represents the mean fitting of the pooled data: *y*=*a*/(1+*e*^−*c*(*x*−*T*)^) with *a*=0.689, *T*=124.187 and *c*=0.0205 (d.f.=8; *R*^2^=0.24). The first aggregate (0°) was excluded from the fitting. (*b*) Angular distribution of the aggregates presented at the end of experiments. 0° is the normalized position of the first aggregate. The solid line represents the mean fitting of the pooled *data*:=*a*/(1+*e*^−*c*(*x*−*T*)^), with *a*=0.619, *T*=174.423 and *c*=0.0195 (d.f.=8; *R*^2^=0.48). The first aggregate (0°) was excluded from the fitting. (*c*) Size of all secondary aggregates (mean number of individuals during the aggregate lifetime) as a function of their angular position from the first aggregate (0°). Logarithmic scale for the *y*-axis. The solid line represents the mean fitting of the pooled data *Y* =2.45*e*^0.012*X*^ (d.f.=193; *R*^2^=0.23).

Second, the relative spatial position of the secondary aggregates was recorded (i.e. relatively to the position of the first aggregate). Regardless of the set-up (see complete statistical data in the electronic supplementary material, table S1), the secondary aggregates were not randomly distributed in the arena (Rayleigh test, 0.6168>*R*>0.2715, *p*<0.0091). Their mean position ranged between 166.6° and 194.2° (electronic supplementary material, table S1). Especially, there is no difference between the positions of the second aggregate in the three set-ups 1Shs, 1Shl and 0Sh (2Sh excluded; Kruskal–Wallis test, KW=3.138, d.f.=2, *p*=0.2082). In addition, the number of stable secondary aggregates observed increased with the distance from the first aggregate (figure 5*a*,*b*). In other words, the probability of the aggregate surviving during the experiments increased with the distance from the first aggregate (figure 5*a*,*b*). Additionally, the size of the secondary aggregates (i.e. the mean number of individuals aggregated inside) increased with the distance from the first aggregate (figure 5*c*). The slope presented in figure 5*c* is significantly non-zero (*F*=54.59, *p*<0.001). The variance of the size of the secondary aggregates increased with the distance from the first aggregate (figure 5*c*; ANOVA test, *F*=8.421, *p*<0.0001).

### 3.4 Synthesis

**Figure 6.**
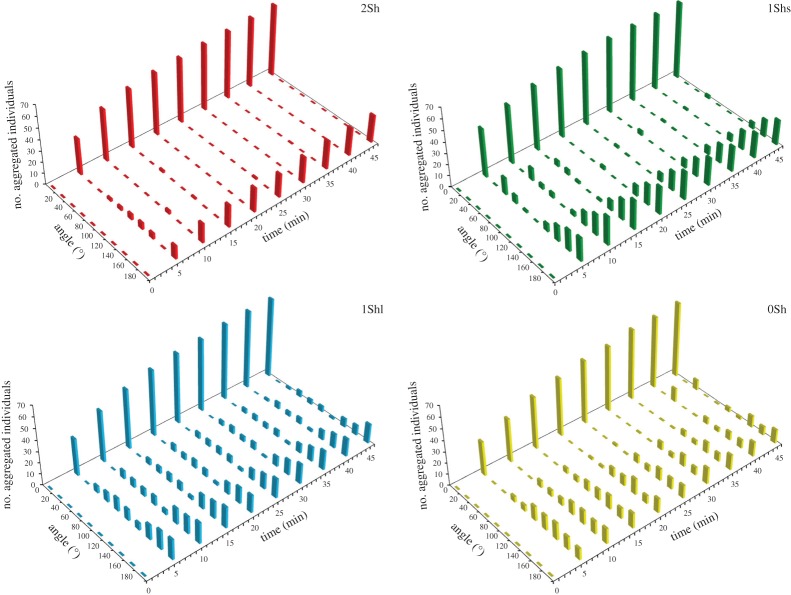
Synthesis of the spatio-temporal distribution of aggregated individuals in the four set-ups. The value 0° is the normalized position of the principal aggregate.

Figure 6 synthesizes the spatio-temporal distribution of individuals in the four set-ups. In particular, we show that the aggregation pattern was similar regardless of the fragmentation of the set-up. We highlight four major components of the process: (i) a fast dynamic; (ii) the formation of several aggregates with at least two stable aggregates (one large aggregate of approx. 70 individuals and one small, of approx. 20 individuals); (iii) a non-random distribution of the aggregates from each other with an increasing probability to stabilize with the inter-aggregate distance; and (iv) a positive relationship between the inter-aggregate distance and the aggregate size.

## 4. Discussion

In the current study, we explored how the trade-off between the environmental heterogeneities and the social mechanisms involved in group cohesion may regulate the group size and spatio-temporal distribution of individuals and aggregates in social species.

First, we stress the characteristics of aggregation in woodlice that have been observed in previous studies, especially its recurrence and its rapidity [12,29,30]. In addition, the formation of a dominant aggregate under one of the two identical shelters offered (2Sh) and in the homogeneous set-up (0Sh) strengthens the demonstration of an inter-attraction between individuals during the aggregation process [12]. These characteristics highlight the significant social component in the aggregation [7,8,12] and support the particular adaptive importance of this behaviour for woodlice [31,32]. Our shelters are favourable places for aggregation because they meet the individual preferences of the woodlice [33]. Therefore, as in many social species, patch selection is the result of the interplay between the response to environmental heterogeneities and the interactions among conspecifics [4,9,12,34].

However, this study shows that aggregation in woodlice is only slightly influenced by the heterogeneity of the environment. Our results indicate that the presence of shelters essentially influences the location of aggregates but not the general features of the aggregation process (occurrence, dynamic, stability, etc.). In other words, a shelter is not necessary for the rapid generation of a large and stable aggregate, but shelter, as a patch of better quality habitat for resting, contributes to the emergence of an aggregation and probably to its stability (see slight disturbance in homogeneous set-up). Other major social factors should influence, much more than heterogeneities, the stability, such as the number of woodlice included in the aggregate [28,35].

Important points emerging from this study are (i) the aggregate-size distribution and (ii) the spatial non-random distribution of aggregates.

Herein, and contrarily to a previous hypothesis and observation in other gregarious arthropods [13,30,35], our study shows that the shelter size and its carrying capacity have low impact on the segregation of individuals into two aggregates of different size. Our results support this for several reasons: first, the aggregates may overflow the shelter limits; second, population splitting is also observed when a large shelter is offered (1Shl set-up), despite 40% remaining available space; and third, populations also spontaneously split in the homogeneous set-up, even without the physical constraints of a shelter.

In addition, the spatial distribution of aggregates from each other is not random, regardless of the set-up. Owing to the preference of individuals for the shelter, the set-ups with two identical shelters diametrically opposed inevitably results in a perfectly symmetrical spatial distribution of the aggregates. However, the results obtained in the homogeneous set-up and in set-ups without a second shelter show that the secondary aggregate resulting from the population splitting is also preferentially formed diametrically opposite the largest aggregate.

Therefore, in our experimental conditions, the heterogeneities of the environment only slightly influenced the spatial distribution both at the individual and group level. In many biological systems, including woodlice, the aggregation process is based on the self-amplification of simple rules at the individual level (i.e. no knowledge of the global structure is required), so that the more individuals engaged in a behaviour, the more the probability that other individuals will exhibit similar behaviour [7–9,11,35–39]. Several direct or indirect interactions based on pheromone concentration or on an encounter rate between conspecifics can be involved in the self-amplification of the process (*positive feedbacks* [7,9]). In gregarious woodlice, the aggregation emergence is based on an increasing probability of joining and a decreasing probability of leaving the group with increasing group size [35]. These mechanisms, resulting in an increasing residence time with group size, are at the basis of the amplification process [35]. Thus, following these simple rules, smaller aggregates should progressively disappear to the benefit of a larger one. In other words, at the local level, a large aggregate should absorb close neighbours and inhibit the development of other aggregates in its immediate vicinity. This is what we observe in our four set-ups with the rapid clearing of emergent aggregates close to dominant aggregates. However, the influence area of an aggregate strongly depends of the range of perception of individuals, so that several aggregates distant enough from each other can coexist. Especially, we highlight such a phenomenon with the increasing probability of observing stable secondary aggregates with increasing distance from the first aggregate and the increasing size of the secondary aggregates with increasing distance from the first aggregate. These patterns resulting from the complex game of spatial competition between distant aggregates are an illustration of the LALI (local activation/ long-range inhibition) model of Gierer & Meinhardt [40] involving scale-dependent feedbacks [21]. The spatial self-organization demonstrated herein with terrestrial isopods is one of the first illustrations of a similar process in a social model. Indeed, if self-organization evidence has been shown in many taxa from unicellular to complex organisms [7,9], few studies have shown such phenomena involved in the spatial organization of animal groups (see distribution in ant cemeteries [18], in mussel beds [15] and also [41]).

Notably, the spatial distribution of aggregates and the aggregate-size distribution in our study are in agreement with the spatial self-organization recorded in many biological and ecological contexts, such as in vegetation patterns [7,21,42–45]. Interestingly, our aggregate-size distribution follows an exponential law close to pure power-law from exponential decay observed for the distribution of group size in herbivore herds or fish schools [46,47] (see the relationship between size-frequency power laws and rank-frequency exponential relations in [48]). Theoretical models on the animal cases show how the group-size distributions result from the basic dynamics of group formation [46,47]. In terrestrial isopods, this dynamic of formation of the aggregate is modulated by density-dependent probabilities of joining and leaving the group [35]. In this view, the experimental patterns observed here for the spatio-temporal distribution of woodlice groups argue in favour of a likely generalizable mechanism to many self-organized species and deserves to be examined in other gregarious species.

In the present experiment, whether the system is at equilibrium is difficult to say. Longer experiments should address the important question of the long-term competition between aggregates and especially the dynamic of their formation/dispersion. Indeed, notably because the probability to leave an aggregate is not null and *negative feedbacks* (such as substrate depletions) can occur [7,9], a stock of moving individuals is always present in the system. Thus, distant aggregates can compete in exploiting this stock of mobile individuals. Additionally, future studies are necessary to understand better the decision of individuals to form secondary aggregates (which is less effective for reducing individual water losses *a priori* [24]).

## Supplementary Material

Electronic Supplementary Materials.pdf
