# Supplementary material for: Regulatory mechanisms of group distributions in a gregarious arthropod
Source: R Soc Open Sci. 2015 Nov 25;2(11):150428. doi: 10.1098/rsos.150428 (PMC4680614; doi:10.1098/rsos.150428)
Supplement: Electronic Supplementary Materials.pdf [file rsos150428supp1.pdf]

Electronic Supplementary Materials

Regulatory mechanisms of group distributions in a gregarious arthropod

Pierre Broly[1,*], Romain Mullier[2], Cédric Devigne[2, 3], Jean-Louis Deneubourg[1]

[1] Unité d'Ecologie Sociale, Université Libre de Bruxelles, Campus de la Plaine, Bruxelles, Belgium; [2] Laboratoire Ecologie & Biodiversité, Faculté de Gestion, Economie & Sciences, UCLILLE, Lille, France; [3] University Lille Nord de France – UDSL, Forensic Taphonomy Unit, F-59000 Lille, France

[*] corresponding author: pierre.broly@icl-lille.fr

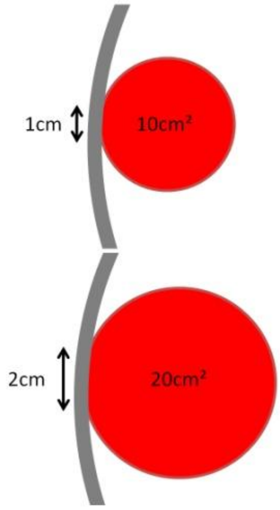

Figure S1. Schematic representation of the small shelter (in the setup 2Sh and 1Shs) and of the large shelter (1Shl).

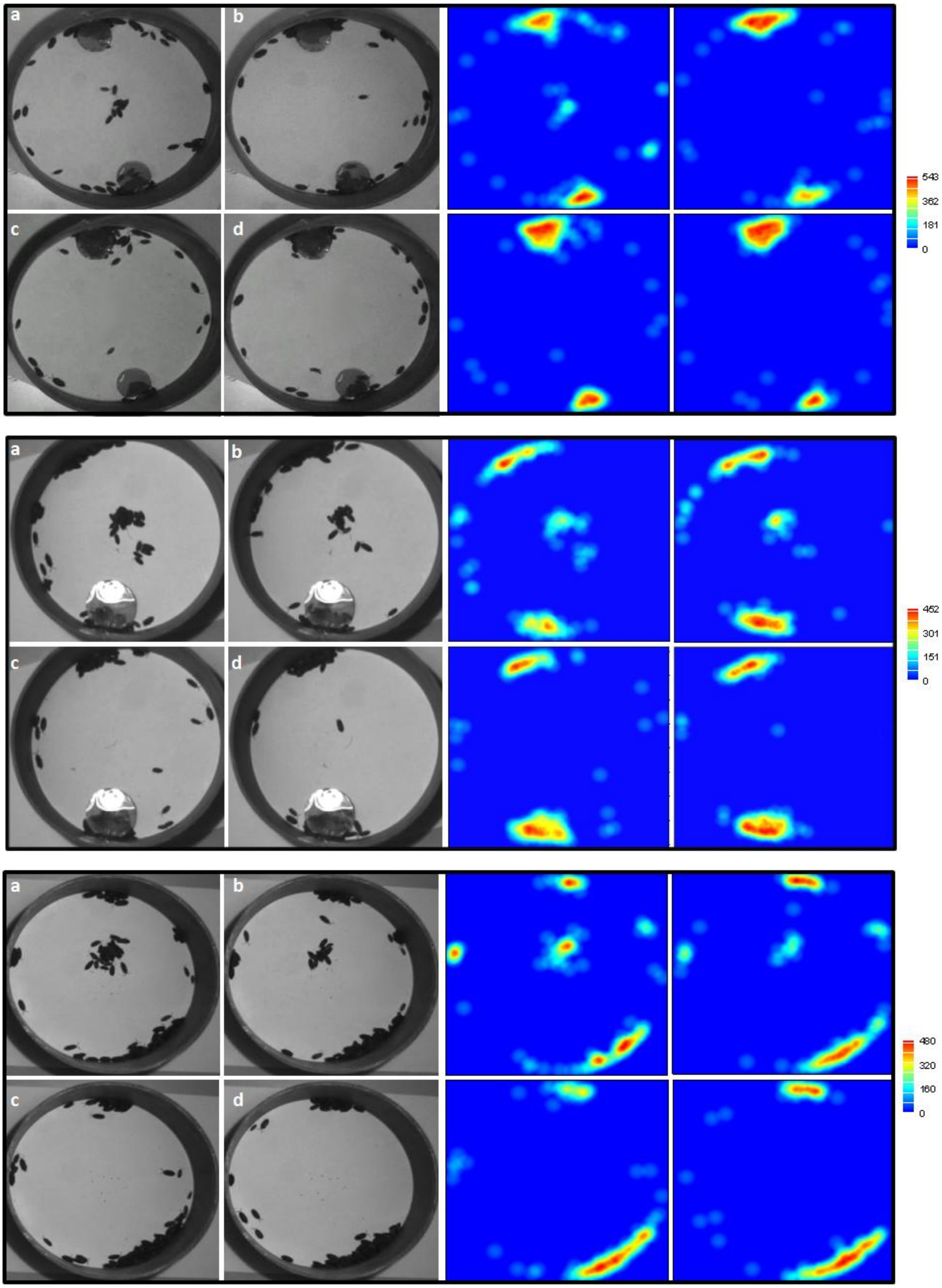

Figure S2. Examples of the aggregation dynamics in the arena with two shelters (top), with one shelter of ø 5 cm (middle) and without a shelter (bottom) at t=5 min (a), t=10 min (b), t=30 min (c) and t=45 min (d) and their representation using Kernel density on the right side (paraboloid function).

Aggregation dynamics

Regardless of the setup used, the global dynamics of aggregation (whatever the spatial localization of individuals) was similar among the conditions: in 10 minutes, 70% of the woodlice were aggregated before reaching a plateau, including between 80 and 90 individuals, until the end of experiments (Tab. 1, Fig. S3a). Only the setup without shelter (0Sh) presented a significantly lower number of aggregated individuals than in the 1Shl setup at the end of the experiments (Fig. S3b; Kruskal-Wallis test, KW= 19.028, df= 3, p< 0.003; Dunn post-test; p<0.001). All other conditions were not significantly different (Dunn post-test; p> 0.05).

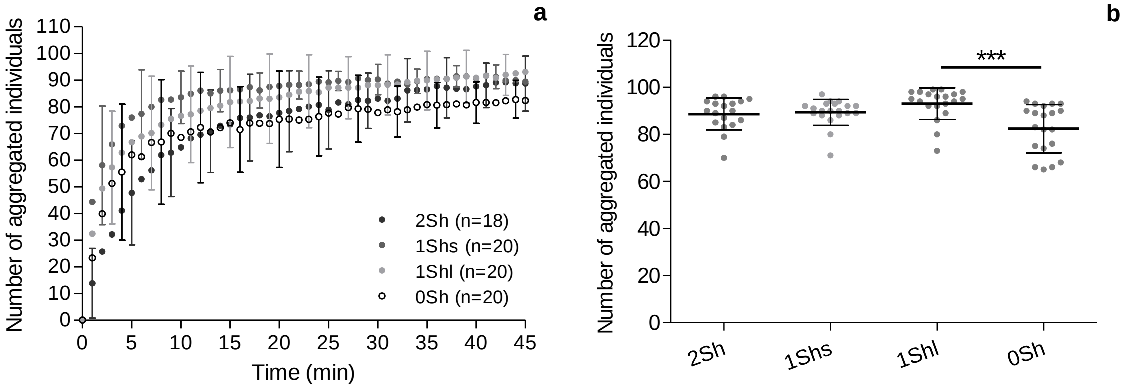

Figure S3a. Dynamic of the total aggregation (total number of individuals aggregated whatever their spatial localization) in the four setups.

Figure S3b. Distribution of the total number of aggregated individuals at the end of the experiment (45th minute). Black lines represent the mean with standard deviation. *** denotes significant difference among the setups.

The dynamics of formation of the aggregates in the four setups are given in Fig. S3c. These dynamics were characterized by a similar pattern: a quick increase in the number of formed aggregates followed by a slow decrease (i.e., certain aggregates disappeared with time). There was an inter-setup difference in the total number of aggregates formed during the experiment (the setup without shelter presents more aggregates than those with 2 shelters; Kruskal-Wallis and Dunn post-test; KW= 15.875, df= 3, p= 0.0012; Fig. S3c), but there was no difference at the end of experiment (Kruskal-Wallis test, KW= 5.947, df= 3, p= 0.1142; Fig. S3c). In more than 75% of the experiments, only two aggregates were still present at the end of the experiments (Tab. 1; Fig. S3c).

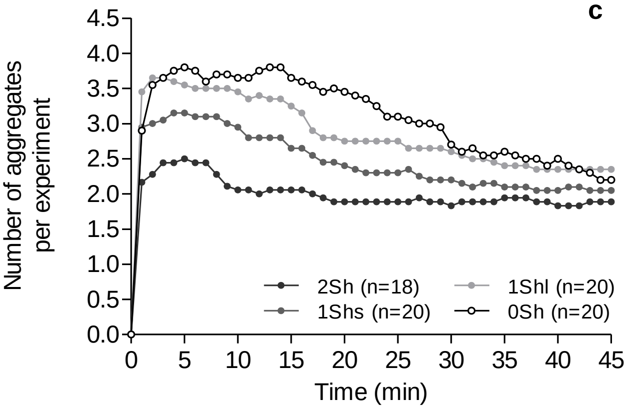

Figure S3c. Mean number of aggregates observed per experiment according to the time in the four setups. At the end of the experiments, the number of aggregates was equal between the setups (Kruskal-Wallis test, KW= 5.947, df= 3, p= 0.1142) and approximated two aggregates (Tab. 1).

The dynamics of aggregation in the first aggregate was followed first during the 45 minutes of the experiment (Fig. 3a). Aggregation was a rapid phenomenon because 50% of the overall population was aggregated in this aggregate after 15 minutes regardless of the setup. Then, the aggregate growth dynamic reached a relative plateau phase. At the end of the experiments, the number of woodlice aggregated herein (approximately 70 individuals) was not significantly different among the four setups (Kruskal-Wallis test, KW= 3.176, df= 3, p= 0.3635; Fig. S3d).

The dynamics of aggregation in the 2nd aggregate (i.e., the second largest aggregate after the 1st aggregate) was also followed (Fig. 3b). As with the 1st aggregate, the aggregation process was fast and reached a relative plateau phase after 15 minutes regardless of the setup used. At the end of the experiments, there was no inter-setup difference in the number of individuals aggregated in the 2nd aggregates (Fig. S3e; Kruskal-Wallis test, KW= 7.642, df= 3, p= 0.0654). The setup with two shelters presented the largest 2nd aggregate (~ 25 individuals) and the setup without shelter the smallest (~ 15 individuals). In more than 75% of the experiments, there was a significant difference between the number of woodlice in the 1st and in the 2nd aggregate at the end of the experiments (Tab. 1; binomial test, p< 0.001).

At last, the other secondary aggregates (≥ 3rd aggregate) formed in the arena presented a highly different pattern due to their weak size, stability and lifetime (Fig. S3f).

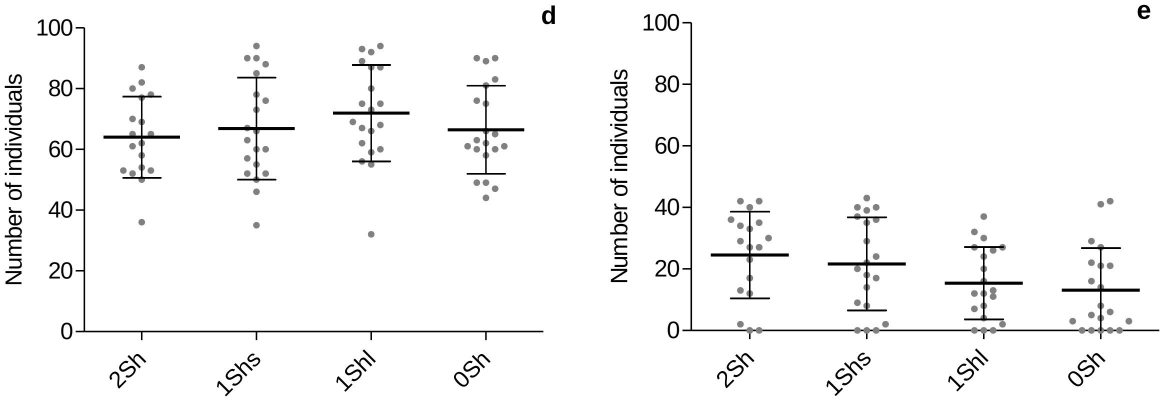

Figure S3d, e. According to the setup, distribution of the number of individuals included in the 1st (f) and in the 2nd aggregate (g) at the end of experiments (45th minute). Black lines represent the mean with standard deviation.

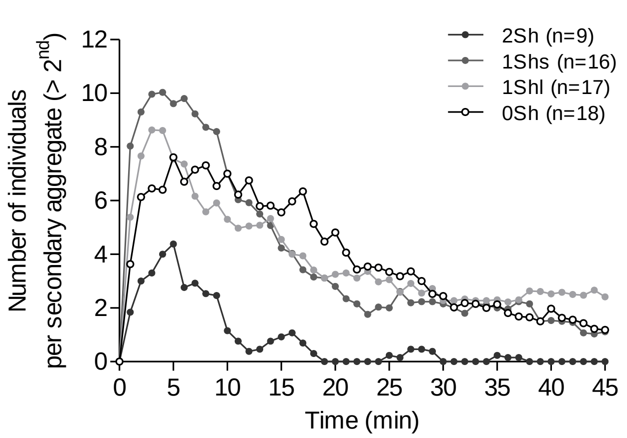

Figure S3f. Inter-setup comparison of the average number of woodlice per secondary aggregates (2nd aggregate excluded) as a function of the time.

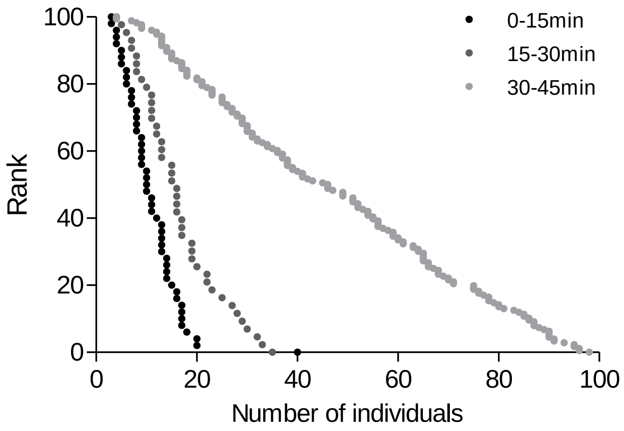

Figure S4. Distribution of three classes of aggregate lifetime according to the maximal aggregate size reached during the experiments. The number of individuals included in the aggregate clearly increased the lifetime of the aggregate.

Spatial conformation of the aggregates

First, the surface area occupied by the 1st aggregates did not differ among the setups at the end of the experiments (Kruskal-Wallis test, KW= 1.837, df= 3, p= 0.6070). Additionally, the surface areas per individual in the 1st aggregate (Fig. S5a) and in the 2nd aggregate (Fig. S5b) did not differ among the setups at the end of experiments (Kruskal-Wallis test, KW= 2.664, df= 3, p= 0.4464 and KW= 2.663, df= 3, p= 0.4465, respectively). However, the surface area per individual decreased with increasing aggregate size (Fig. 5c), and, for each setup, the surface area per individual was significantly lower in the 1st aggregate than in the 2nd aggregate (Mann-Whitney test, 2Sh: n= 18; U=39, p= 0.0107; 1Shs: n= 20; U=35, p=0.002; 1Shl: n= 20; U=45, p=0.0026; 0Sh: n=20; U=22, p<0.0001). In other words, the number of individuals included in the aggregate affected the spatial spread of the aggregates, but the presence of a shelter (regardless of its diameter) (Fig. S5a, S5b, S5c) did not affect the spread of the aggregates.
In addition, the filling of the shelter containing the 1st aggregate was calculated at the end of experiments to estimate the shelter saturation (Fig. S5d). The shelters of 3.5 cm diameter in setup 2Sh and 1Shs exhibited filling to approximately 90% of their total carrying capacity, whereas the shelter of 5 cm diameter exhibited filling to approximately 60%. This difference was significant (Kruskal-Wallis test, KW= 26.152, df= 3, p< 0.001). Additionally, the surface area of the aggregate overflow from the shelter limits represented more than 40% of the total surface area of the aggregates under the small shelters of 3.5 cm diameter, whereas the shelter overflow represented only 20% in aggregates under the large shelter of 5 cm diameter (Fig. S5e). This difference was significant (Kruskal-Wallis test, KW= 23.783, df= 3, p< 0.001).

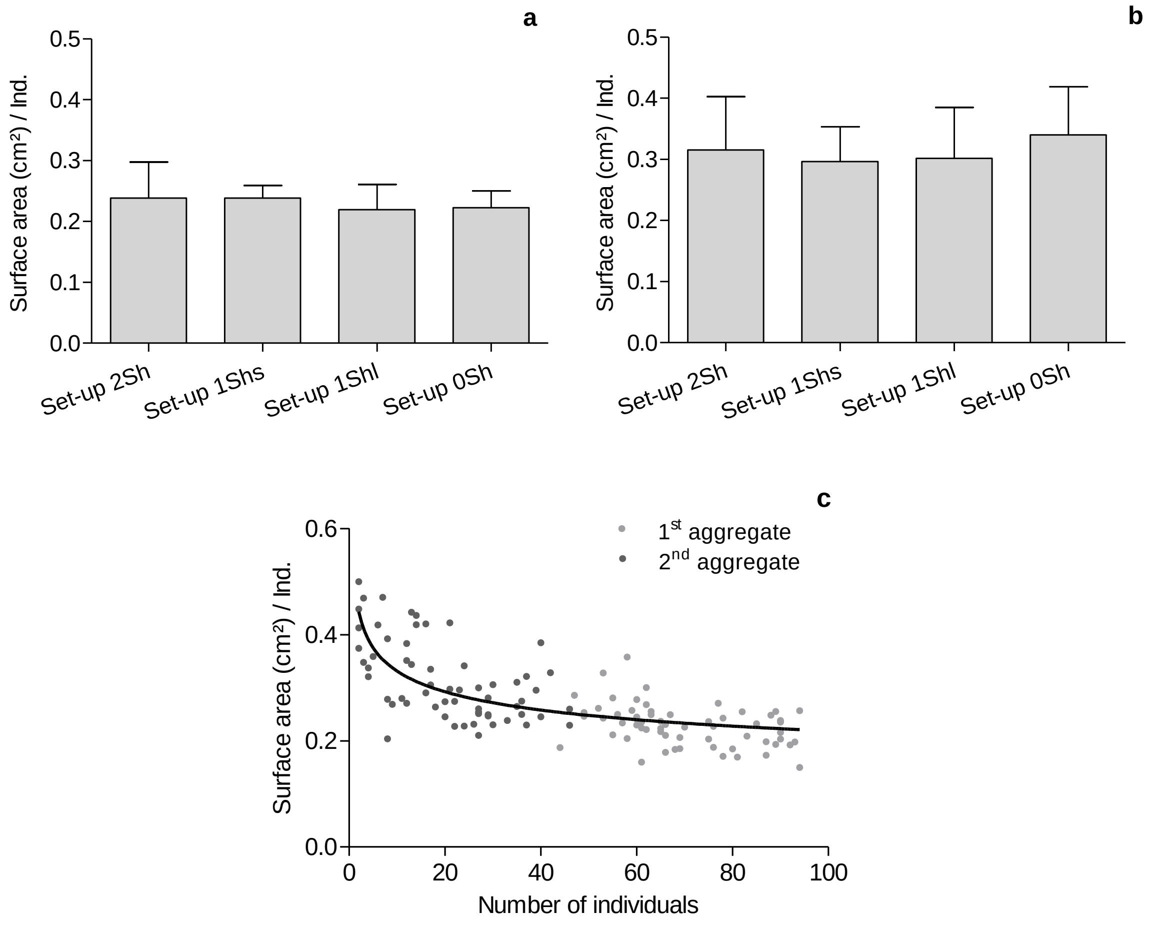

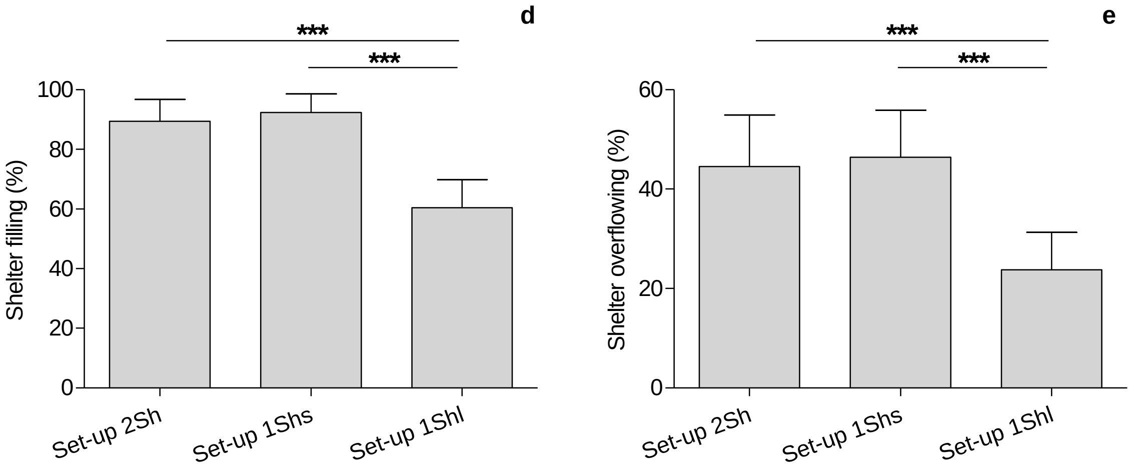

Figure S5a. Surface area (in cm²) per individual of the 1st aggregate in the four setups, at the end of experiments. There was no significant difference among the setups (Kruskal-Wallis test, KW=2.664, df= 3, p=0.4464).

Figure S5b. Surface area (in cm²) per individual in the 2nd aggregate in the four setups, at the end of experiments. There was no significant difference among the setups (Kruskal-Wallis test, KW=2.663, p= 0.4465).

Figure S5c. Surface area (in cm²) per individual (S) in the 1st and in the 2nd aggregate as a function of the aggregate size (N). The solid line represents the mean fitting of the pooled data $S= aN^b$ with a= 5.02 (+/- 0.0.022) and b= -0.18 (+/- 0.0137) (df= 122; R^2= 0.5463).

Figure S5d. Percentage of filling of the shelter occupied by the principal aggregate at the end of experiments according to the setup. There was a significant difference in the shelter filling (Kruskal-Wallis test, KW= 26.152, p< 0.001); *** represents inter-experimental differences (Dunn post-test, p< 0.001).

Figure S5e. Surface area of the principal aggregate overflow from the shelter limits (expressed in percent of the total surface area of the aggregate at the end of the experiments) in the three setups with shelter(s). There was a significant difference in the shelter overflow (Kruskal-Wallis test, KW= 23.783, p< 0.001); *** represents inter-experimental differences (Dunn post-test, p< 0.001).

Table S1. Summary of the statistical tests on the secondary aggregate distributions in the four setups.

	Secondary aggregates		
	Number of aggregates observed	Rayleigh's test	Mean angle (°) IC: 95%
**2Sh**	29	R= 0.4874 p< 0.001	166.6 (127.2 to 206)
**1Shs**	46	R= 0.6168 p< 0.001	185.79 (169.3 to 204.2)
**1Shl**	55	R= 0.3806 p< 0.001	182.97 (147.6 to 218.3)
**0Sh**	63	R= 0.2715 P= 0.0091	194.19 (148 to 240.4)
